# Changes in Parents’ Home Learning Activities With Their Children During the COVID-19 Lockdown – The Role of Parental Stress, Parents’ Self-Efficacy and Social Support

**DOI:** 10.3389/fpsyg.2021.682540

**Published:** 2021-07-29

**Authors:** Elisa Oppermann, Franziska Cohen, Katrin Wolf, Lars Burghardt, Yvonne Anders

**Affiliations:** ^1^Chair of Early Childhood Education, University of Bamberg, Bamberg, Germany; ^2^Department of Early Childhood Education, University of Education Freiburg, Freiburg, Germany

**Keywords:** COVID-19, home learning environment (HLE), parental stress, family stress model, social support, parental self-efficacy

## Abstract

As a result of the abrupt closures of daycare centers in Germany due to the COVID-19 pandemic, parents’ ability to provide learning opportunities at home became all the more important. Building on the family stress model, the study investigates how parental stress affected changes in parents’ provision of home learning activities (HLA) during the lockdown, compared to before the lockdown. In addition, the study considers parental self-efficacy and perceived social support as protective factors that may play important roles in disrupting the negative effects of stress. Data stems from a nation-wide survey of 7,837 German parents of children ages 1–6 years, which was conducted in Spring 2020 during the first wave of COVID-19 infections and at a time of strict restrictions in Germany. Results revealed that parental stress was negatively related to changes in the provision of HLA. Parental self-efficacy and an intact social support system were protective of parental stress during the lockdown. Additionally, parental self-efficacy and – to a larger extend – perceived social support interacted with parental stress in the relation to changes in the provision of HLA. Specifically, self-efficacy and perceived social support acted as protective factors that buffered the negative influence of stress on parents’ ability to provide educational activities for their children at home. These results have important implications for supporting families with young children during challenging times, such as the COVID-19 pandemic and the temporary closure of daycare centers.

## Introduction

As a response to the COVID-19 pandemic, Germany – among many other countries – implemented nation-wide restrictions to slow the spread of the virus in Spring 2020. These restrictions included the closure of daycare centers and schools^1^ as well as all other educative supporting services directed to children, the prohibition to visit playgrounds as well as strict social distancing measures, e.g., no contact with more than one person from outside one’s household^2^. This led to a challenging situation for families with young children (Andresen et al., 2020; Huebener et al., 2021). Children stayed at home all day and parents had to provide early education and care while simultaneously having to meet all other demands, e.g., occupation, household. The availability of stimulating home learning activities (HLA) is crucial for children’s development (Melhuish et al., 2008; Anders et al., 2012) – even more so when institutional education is unavailable, and children rely on their parents to support their learning and development at home. For this reason, we were especially interested in the way parents coped with these challenging times and how the lockdown changed the provision of HLA.

Research shows that parents’ ability to provide HLA can be impaired by parental stress, which might also apply to stress resulting from the COVID-19 lockdown (Gershoff et al., 2007). Building on the family stress model (Conger et al., 1992; Masarik and Conger, 2017), the present study examines how parental stress during the lockdown was related to changes in parents’ provision of HLA. Moreover, extending the family stress model, the study considers parental self-efficacy and perceived social support as protective factors that may play important roles in disrupting the negative effects of stress. A better understanding of the processes linking parental stress and HLA, as well as the potential benefit of protective factors in this relation, is essential to promoting children’s learning and development during difficult times, such as a the COVID-19 pandemic.

### Home Learning Environment

The home learning environment constitutes the first and most influential developmental context for children (Bronfenbrenner and Morris, 2006), which has greater effects on child outcomes than any other context (Melhuish et al., 2008). The quality of the home learning environment is a broad concept that encompasses the availability of resources (i.e., structural characteristics such as learning materials and family income), parents’ beliefs that influence the provision of learning opportunities (such as parental educational aspirations), and the quantity and quality of parent-child interactions that promote learning (Anders et al., 2012; Kluczniok et al., 2013; Lehrl et al., 2020). These parent–child interactions take place during HLA, e.g., joint book reading or solving puzzles together, which are essential for children’s learning and development because they provide children with everyday learning opportunities (Kluczniok et al., 2013). The importance of HLA for child development is widely empirically documented (Foster et al., 2005; Melhuish et al., 2008; Anders et al., 2012; Lehrl et al., 2020; Liang et al., 2020). Parents’ ability to provide everyday HLA may thus be particularly crucial at times when institutional early childhood education is not available, such as during the closure of daycare centers in Spring 2020. One aim of the present study was to investigate changes in HLA during the COVID-19 lockdown compared to before the lockdown. One may assume that parents increased the amount of HLA during the closure of daycare centers – also because parents spent more time with their children. However, it is important to keep in mind that the abrupt closure of daycare centers created a stressful situation for parents, who still had to meet all other demands, including their occupations, household, and potential care and home-schooling of other children. Thus, even if parents intended to compensate for the missing institutional education, increased parental stress may have impaired their ability to provide more HLA for their children.

### Family Stress Model: Relation Between Parental Stress and Changes in HLA

The family stress model illustrates that stressors, such as financial problems and problematic living conditions, increase parental stress and – through that – negatively influence parenting behavior (Conger et al., 1992; Kotchick et al., 2005; McConnell et al., 2011; Masarik and Conger, 2017). The assumed relations of the family stress model have also been applied to the provision of HLA (Gershoff et al., 2007; Raver et al., 2007; Bendickson, 2020). For instance, Gershoff et al. (2007) showed that economic hardship among US parents of 6–7-year-old children was associated with increased parental stress and poorer parenting behavior, including less provision of cognitively stimulating parent–child activities. More recently, a study based on data from nine different European countries showed that material deprivation was negatively associated with parental wellbeing and parents’ provision of HLA (Wolf et al., 2019).

In the present study, we focus on parents’ perceived stress, which is defined as psychological distress that occurs as a reaction to external risk factors at a given time (Randall and Bodenmann, 2013). Perceived psychological stress thereby reflects individual parents’ appraisal of environmental stressors, which they feel are taxing or exceeding their resources for coping (Lazarus and Folkman, 1984). It is important to distinguish psychological stress from related concepts, such as burnout or depression, which are more long-term clinical conditions. Parental stress can be caused by a number of stressors (Masarik and Conger, 2017), such as financial strain, as proposed by the family stress model (Gershoff et al., 2007; Green et al., 2007; Scaramella et al., 2008; Stewart and Cooper, 2013). Although many studies referring to the family stress model focused on financial problems as a main stressor, there is evidence that other factors may also cause parental stress, including problematic housing conditions, e.g., living in problematic neighborhoods (Ross, 2000; Scaramella et al., 2008), or in houses that are too small for the family (Ross, 2000), and work-related problems, e.g., unemployment (Algarvio et al., 2018). In addition, environmental influences and hazardous events could act as stressors, such as the COVID-19 pandemic (Ravens-Sieberer et al., 2021). However, a recent study showed that health concern was not a significant predictor of perceived stress among parents of children aged 2–14 years in northern Italy, which was one of the European regions most affected by the first wave of COVID-19 infections (Spinelli et al., 2020). Instead, those parents who reported to have difficulty meeting all their demands were most stressed (Spinelli et al., 2020). This suggests that the increase in parental stress in Spring 2020, which has also been documented among German parents by Huebener et al. (2021), was not mainly due to health concerns but rather a consequence of the lockdown. There are two ways through which the lockdown may have increased stress for parents: first, the lockdown may have intensified existing stressors, such as the stress caused by living in inadequate housing, e.g., apartments/houses that are too small for the family size, which is likely to increase conflict at home when more family members stay at home and outside activities, like the use of playgrounds, are prohibited. Second, it may have led to additional stressors, such as unemployment or short-time leave, leading to increasing financial problems; or working from home while having to care for small children. These (additional) stressors during the COVID-19 lockdown likely increased parental stress – which, based on the assumptions of the family stress model, should lead to less HLA. In this regard, one may assume that HLA decreases linearly with increasing parental stress: The more parents are stressed, the less cognitive and emotional resources they may have to offer stimulating HLA for their children. Alternatively, one may argue that parents can deal with some stress until a certain threshold is reached, at which point parents feel overwhelmed by the stress, leading to a non-linear decrease in HLA (threshold hypothesis). A non-linear relation between stress and behavioral outcomes has been documented for stress and depression (Rudolph and Flynn, 2007) as well as for cumulative risk and child development (Evans et al., 2013), but the threshold idea has rarely been applied to parents and it has not been investigated among parents during the COVID-19 pandemic.

### Protective Factors

The theoretical and empirical research literature suggests that external stressors result in parental stress (Ross, 2000; Scaramella et al., 2008; Masarik and Conger, 2017), which results in non-optimal parenting behavior, including HLA (Gershoff et al., 2007; Wolf et al., 2019; Bendickson, 2020). Yet, this is not the case for every household. Some parents seem to be more “resilient” than others, which suggests that there may be internal and external protective factors that disrupt this negative circle. In fact, the family stress model proposes that protective factors may interact with parental stress and reduce the negative impact of stress on parenting practices (Masarik and Conger, 2017). The present study focuses on two factors that have been demonstrated to reduce stress and improve parenting behavior, namely parental self-efficacy beliefs (Bojczyk et al., 2018) and social support (McConnell et al., 2011).

#### Parental Self-Efficacy Beliefs

Parental self-efficacy beliefs are defined as parents’ beliefs that they can promote their child‘s development and their environment toward positive child outcomes (Ardelt and Eccles, 2001). The construct is based on Bandura’s (1977) social cognitive theory, describing self-efficacy as a primary source of human motivation and action. In line with this, empirical findings show that higher parental self-efficacy beliefs are associated with more HLA (Jones and Prinz, 2005; Peacock-Chambers et al., 2017; Bojczyk et al., 2018) and better child outcomes, such as child adjustment (Bojczyk et al., 2018) and lower problem behavior (Bandura, 1997; Jones and Prinz, 2005). Moreover, previous findings show that parents with higher parenting self-efficacy beliefs cope better with difficult parenting situations (Bojczyk et al., 2018) and report lower parental stress (Bloomfield and Kendall, 2012; Albanese et al., 2019 for an overview). This may be because parents who are confident in their ability to support their children’s learning and development may see difficult parenting situations as challenges rather than problems and face these challenges with lower negative emotional arousal or stress (Jerusalem and Mittag, 1995). In addition to its influence on perceived stress and parenting practices, parental self-efficacy has been discussed as a moderator of the relation between parental stress and parenting practices (Albanese et al., 2019). Specifically, parents’ confidence in their ability to handle even difficult parenting situations may help them cope with the stress and promote their child’s learning, thus buffering the negative influence of stress on HLA. Previous studies have documented the moderating function of parental self-efficacy in the relation between marital stress and infant-mother attachment quality (Cassé et al., 2016) and in the relation between parental distress and parenting style and consistency (Rominov et al., 2016). The potential moderating role of parental self-efficacy in the relation between parental stress and changes in HLA, however, has not been examined yet.

#### Perceived Social Support

Social support is the perception that one is part of a social network that provides psychological and material resources intended to benefit a person’s ability to deal with stress (Cohen, 2004). Social support may take different forms, including emotional support (e.g., empathy, caring, concern, affection, and reassurance), informational support (e.g., advice, guidance, and suggestions), and instrumental support (e.g., provision of material goods or services, such as helping with the household, caring for the children). Parents’ perceived social support has been shown to help parents cope with stress (Östberg and Hagekull, 2000; McConnell et al., 2011; Parkes et al., 2015; Masarik and Conger, 2017). As it can be assumed that the COVID-19 lockdown resulted in higher stress for parents, perceived social support may have acted as a central protective factor for parents’ perceived stress. At the same time, keeping social contacts during the lockdown may have been increasingly challenging due to social distancing measures. Thus, parents’ perception of the support that they can rely upon during the lockdown may have been particularly relevant for their wellbeing – even more so during the lockdown than during ‘normal’ times.

In addition, perceived social support has been linked to parenting practices (McConnell et al., 2011), including HLA (Green et al., 2007; Bendickson, 2020). For instance, parents with more social support showed a higher frequency of positive parent–child activities (Green et al., 2007), higher parental warmth (Izzo et al., 2000), and less ineffective parenting (McConnell et al., 2011). Moreover, previous studies documented that perceived social support functions as a moderator of the relation between parenting stress and parenting practices by enhancing parents’ resilience in difficult situations (Kotchick et al., 2005; McConnell et al., 2011). The COVID-19 lockdown can be considered as a particularly difficult situation and perceived social support may thus have played a similar role: Parents with higher perceived social support may have been better able to cope with the stress they experienced, which might have buffered the assumed negative effect of stress on changes in HLA during the COVID-19 lockdown.

### This Study

Based on the assumptions of the family stress model (Masarik and Conger, 2017), the present study examined the influence of parental stress on changes in HLA during the COVID-19 lockdown. Moreover, extending the family stress model, we investigated the role of parental self-efficacy and perceived social support as potential protective factors. We controlled for covariates that are typically associated with HLA and parental stress, specifically parents age, gender, parental education (McConnell et al., 2011; Parkes et al., 2015; Wolf et al., 2019) as well as characteristics that may also affect parental stress during the COVID-19 lockdown (e.g., working from home, being a single parent).

We investigated the following research questions:

(1) How do stressors and perceived parental stress influence changes in HLA during the lockdown?
  (a) We expect that stressors are positively associated with perceived parental stress during the lockdown (H1a).
  (b) We hypothesize that parental stress is negatively associated with changes in HLA (H1b).
  (c) We test for a non-linear relation between stress and changes in HLA (threshold hypothesis). As there are too few previous findings, we explore the nature of the relation between stress and changes in HLA.

(2) How are parental self-efficacy and perceived social support related to parental stress and changes in HLA?
  (a) Parental self-efficacy and perceived social support are negatively associated with perceived stress (H2a).
  (b) Parental self-efficacy and perceived social support are positively associated with changes in HLA (H2b).
  (c) Parental self-efficacy and perceived social support interact with perceived stress in predicting changes in HLA (H2c).

## Materials and Methods

### Sample

Data for this study stems from in a nation-wide cross-sectional online survey in Germany, which was specifically designed to examine the effects of the abrupt closures of daycare centers and the strict regulations due to the COVID-19 pandemic on German families with young children (Cohen et al., 2020). To our knowledge, this was one of the first studies to assess the situation of families with children ages 1–6 years during the first wave of COVID-19 infections and at a time of strict restrictions in Germany in Spring 2020. Parents of children who attended daycare before the closures were invited to participate in the study between April 9th and May 24th 2020. Participants were recruited using convenience sampling starting with personal contacts, online blogs, social media and mailing lists of large non-profit organizations, foundations, and daycare providers. Altogether 9,343 parents participated in the survey. As the present study investigates the effects of the closures of daycare centers on parental stress and HLA, we excluded those cases where children did not attend daycare at all before the pandemic (*n* = 779) and those cases where children did attend daycare at the time of data collection despite the nation-wide closures (*n* = 727)^3^. The final dataset for our analyses consisted of *N* = 7,837 parents of children ages 1–6 years (*M* = 4.20, *SD* = 1.38) from all 16 federal states of Germany. The participants were on average 37.10 years old (*SD* = 4.50), 88.3% were female. Parental education was coded into three levels: *low* which corresponds to ISCED levels 0–2 (lower secondary school education and below; see International Standard Classification of Education; UNESCO, 2011), *medium* which corresponds to ISCED levels 3–5 (upper secondary school education to short-cycle tertiary education) and *high* which corresponds to ISCED levels 6 and 7 (Bachelor degree or above). In the sample, 74.2% of parents had a high educational level, 25.0% had a medium educational level and 0.8% had a low educational level. At the time of data collection, 72.5% of the parents were employed (20.6% in full time, 43.6% in part time and 8.4% in short-time work with temporarily reduced hours), compared to 78.3% who were employed before the COVID-19 pandemic.

Written informed consent was given by the participants. Participants were informed that they could stop the survey at any time without any disadvantage. The study abided APA ethical guidelines on conducting studies with human participants. No formal approval from a governing or institutional review board was required for the study (see guidelines provided by the German Research Foundation for the social sciences^4^).

### Measures

#### Central Variables

##### Stressors

The following potential stressors were assessed through single items: financial problems, problematic housing situations, work-related problems, COVID-related health worries, conflict with partner, and conflict with family. Parents were asked whether these stressors occurred in the last weeks and if so, how burdensome they perceived them, ranging 1 (*did not occur*), 2 (*a bit burdensome*) to 5 (*very burdensome*).

##### Parental stress

Parental stress was assessed with four items where parents indicated their agreement with different statements of psychological distress ranging 1 (*totally disagree*) to 4 (*totally agree*). Sample item: “I feel overwhelmed with all the demands I have to meet.” Reliability was good (α = 0.85).

##### Changes in home learning activities (HLA)

Changes in parent–child-activities in the home were measured with reference to the time before the lockdown. Specifically, parents were asked to indicate if they do a certain activity *much less* (1), *less* (2), *slightly less* (3), *same* (4), *slightly more* (5), *more* (6), or *much more* (7) than before the lockdown. The scale consists of nine items, representing activities in the domains numeracy, reading, creative and practical activities (see Supplementary Appendix Table A1). The items were adapted from HLA items used in the German National Educational Panel Study (NEPS) (Blossfeld et al., 2011). Such global HLA measures were used in several German and European studies (e.g., EPPSE, see Sylva et al., 2014) and findings confirmed that these measures are predictive of child development (NEPS: Relikowski et al., 2015; EPPSE: Melhuish et al., 2008; Sylva et al., 2014). Reliability was good (α = 0.84).

##### Parental self-efficacy

Parental self-efficacy was measured using a five-item scale ranging from 1 (*totally disagree*) to 4 (*totally agree*). The measure focused on parents’ general confidence in supporting their children’s development and was thus appropriate for a range of child ages. The scale was developed by Schünke et al. (in preparation) based on an established instrument by Kliem et al. (2014). Sample item: “I have all the skills necessary to be a good mother/father.” Reliability was acceptable (α = 0.79).

##### Perceived social support

Perceived social support was assessed with regard to parents’ report on how often they can rely upon someone to give them emotional and informational support. The scale consisted of four items ranging 1 (*never*) to 5 (*always*), sample item: “Can you rely upon someone to give you advice with problems?” (see Supplementary Appendix Table A2 for the item wordings of the entire scale). Reliability was good (α = 0.89).

#### Covariates and Family Characteristics

All covariates were assessed through parental report. These included (1) children’s age; (2) the number of children living in the household that are 1–6 years old; (3) single parent; (4) private childcare (non-institutional), i.e., anyone outside the household taking care of their child/children for any number of hours, such as grandparents, friends, babysitters; (5) working from home, i.e., whether parents who were employed at the time of data collection currently worked from home and (6) whether both partners were working (part-time or full-time).

### Statistical Analyses

First, descriptive results and bivariate correlations between the observed variables were computed. To analyze whether data on our variables of interest were systematically missing, we conducted missing data analyses with all cases using Little (1998) test of missing completely at random (MCAR). MCAR test results showed no systematic missingness in our continuous variables of interest (i.e., financial problems, problematic housing situation, work related problems, perceived stress, perceived social support, parental self-efficacy and HLA); χ^2^ = 251.53, *df* = 244, *p* = 0.357. To make full use of the data, we applied the full information likelihood method in all our analyses to answer our research questions (FIML). FIML in conjunction with the robust maximum likelihood estimator (MLR), has been found to result in unbiased parameter estimates even with a high percentage of missing data (Enders, 2001; Shin et al., 2009).

The first research question regarding the relation between stressors, perceived parental stress and changes in HLA was investigated using multivariate regression analyses in Mplus (Version 8.3; Muthén and Muthén, 1998–2012). To test for a non-linear relation between parental stress and changes in HLA (threshold hypothesis), we visually examined this relation using locally estimated scatterplot smoothing (LOESS) in ggplot in R (Wickham, 2016; RStudio Team, 2020). Our second research question regarding the role of protective factors in the relation between stress and changes in HLA was investigated using path analyses. This was the most parsimonious approach and it also allowed us to directly compare the effects of self-efficacy and perceived social support on stress and changes in HLA. Model fit was assessed with reference to the Yuan–Bentler scaled χ^2^ (YB χ^2^, mean-adjusted test-statistic robust to non-normality), the root mean square of approximation (RMSEA), the comparative fit index (CFI), the Tucker and Lewis index (TLI), and the standardized root mean residual (SRMR) values using the criteria suggested by Hu and Bentler (1999). CFI and TLI values greater than.95, RMSEA values lower than 0.06, and SRMR lower than 0.08 were accepted as indicators of a good model fit (Hu and Bentler, 1999). As the method to test statistical interaction effects (hypothesis 2c) depends on the nature of the relation between stress and changes in HLA (RQ1), we report the exact analyses to test hypothesis 2c below.

## Results

### Descriptive Statistics and Changes in HLA

Descriptive results revealed that parental stress scores were slightly above the theoretical mean of 2.5, indicating that parents were rather stressed (*M* = 2.70, *SD* = 0.72). With regard to HLA, parents, on average, reported to provide more HLA compared to before the lockdown: The mean of 4.96 was closest to the response format “slightly more” (see Table 1 for descriptive results of the study variables). More detailed examination of the item-specific frequencies showed that the largest increases could be documented in activities related to crafting and arts (e.g., painting), followed by motion play (e.g., running, playing tag, hide, and seek) and music/dancing (see Supplementary Appendix Table A1). A smaller increase in the frequency of activities was found for the domains literacy (e.g., reading, learning rhymes or poems) and math/numeracy (e.g., sorting and classifying objects or construction games). However, there was considerable variance across all items, indicating that – although the average score showed an increase in HLA – some parents reported to do (much) less and some parents (much) more activities than before the lockdown.

**TABLE 1:** Descriptive statistics.

	*N*	*M*/%	*SD*	Min	Max
Age of child in years	7034	4.20	1.38	1.00	6.00
No. of children ages 1–6	7801	1.53	0.60	1.00	4.00
Single parent in %	7828	4.2%		0.00	1.00
Private childcare in %	4456	22.1%		0.00	1.00
Working from home in %	5644	73.0%		0.00	1.00
Both partners working in %	7482	65.8%		0.00	1.00
Financial problems	7007	2.17	1.19	1.00	5.00
Problematic housing	6997	2.15	1.14	1.00	5.00
Work-related problems	6992	3.03	1.28	1.00	5.00
COVID-related health worries	6999	2.62	1.22	1.00	5.00
Conflict with partner	6981	2.60	1.14	1.00	5.00
Conflict with family	6994	2.29	1.11	1.00	5.00
Parental stress	7412	2.70	0.72	1.00	4.00
Changes in HLA	6903	4.96	0.84	1.00	7.00
Parental self-efficacy	7412	3.23	0.44	1.00	4.00
Perceived social support	7020	3.33	1.02	1.00	5.00

*The sample size for parents’ reports on whether they were working from home was comparatively small because this information was only obtained from parents who were employed at the time of data collection. Please note that private childcare refers to non-institutional care, i.e., anyone outside the household taking care of the child/children for any number of hours, including grandparents, friends, babysitters.*

As the sample included a wide range of children’s ages (1–6 years), we additionally examined whether the descriptive statistics differed between children’s age groups (ages 1–2, 3–4, and 5–6). Descriptive results showed several differences between the age groups on our covariates and stressors, such as fewer children and lower percentages of single parents among parents of 1–2-year-olds but also more work-related problems (see Supplementary Appendix Table A3). Parents of older children reported slightly fewer problems with their housing situation, but more conflict with the partner and family. The descriptive statistics of our main variables of interest, namely parental perceived stress, changes in HLA, parental self-efficacy and perceived social support, were very similar in the three age groups. The only two notable differences were that parents of children ages 5–6 years reported a lower increase in HLA compared to parents of children ages 1–2 years and parents of children in the youngest age group felt slightly more self-efficacious than parents of children in the other two age groups.

Bivariate correlations between the study variables are displayed in Supplementary Appendix Table A4. Results showed that all stressors were significantly correlated with parental stress. There was a small negative correlation between some of the stressors and changes in HLA, indicating that stressors relate to a lower increase in activities. The correlation between parental stress and changes in HLA was also negative and slightly larger. Parental self-efficacy and perceived social support were negatively related to the stressors as well as to parental stress. Both, self-efficacy beliefs and perceived social support positively correlated with changes in HLA: the parent-reported change in HLA compared to before the lockdown was positively related to parents’ self-efficacy beliefs and perceived social support.

### RQ1: Associations Between Stressors, Perceived Parental Stress and Changes in HLA

We investigated the associations between the stressors and perceived parental stress during COVID-19 using hierarchical linear regression analyses. The first regression model, which included only covariates predicting parental stress (see Table 2, Model 1), showed that the effects were small and the model only explained 3% of the variance in parental stress. The stressors were added in Model 2, which explained considerably more variance in parental stress than Model 1 (Δ*R*^2^ = 0.28). Results largely supported our hypothesis 1a, stating that stressors are positively related to perceived parental stress. The strongest predictors of parental stress were work-related problems, conflict with the family, and conflict with the partner. Financial problems and COVID-19 related health worries did not significantly predict parental stress when other stressors were accounted for.

**TABLE 2:** Regression results for stressors predicting perceived stress.

	Model 1			Model 2		
		
	β	*SE*	*p*	β	*SE*	*p*
***Covariates***						
Gender (0 = female)	–0.02	0.01	0.093	–0.02	0.01	0.097
Age in years	0.02	0.01	0.152	0.01	0.01	0.203
Education level	–0.02	0.01	0.151	–0.02	0.01	0.188
Age of child in years	–0.06	0.01	0.000	–0.04	0.01	0.001
No. of children ages 1–6	0.08	0.01	0.000	0.07	0.01	0.000
Single parent (0 = no)	0.01	0.01	0.348	0.03	0.01	0.025
Private childcare (0 = no)	–0.03	0.02	0.033	–0.03	0.01	0.049
Working from home (0 = no)	0.15	0.02	0.000	0.12	0.01	0.000
Both partners working (0 = no)	0.07	0.01	0.000	0.06	0.01	0.000
***Stressors***						
Financial problems				0.01	0.01	0.268
Problematic housing				0.14	0.01	0.000
Work-related problems				0.22	0.01	0.000
COVID-related health worries				0.03	0.01	0.002
Conflict with partner				0.18	0.01	0.000
Conflict with family				0.20	0.01	0.000
*R* ^2^	0.03	0.01	0.000	0.31	0.01	0.000

**N* = 7,837.*

To test hypothesis 1b, we investigated the relation between parental stress and changes in HLA using hierarchical regression analyses. The first regression model included only covariates and stressors predicting changes in HLA (see Table 3, Model 1). Results showed that some of the covariates and stressors were negatively related to changes in HLA, however, the effect sizes were small, and the model only explained 3% of the variance in changes in HLA. In a second step, we included perceived parental stress as a predictor of changes in HLA. Supporting our hypothesis 1b, parental stress was negatively related to changes in HLA: The more stress parents reported, the lower the increase in HLA (see Table 3, Model 2). Perceived parental stress was the strongest predictor of changes in HLA and the amount of explained variance increased to 5%. Moreover, some of the stressors’ direct effects on changes in HLA became non-significant when perceived parental stress was accounted for, indicating that parental stress may mediate the relations between stressors and changes in HLA. Additional tests for indirect effects confirmed this: Those stressors that had a significant effect on parental stress also showed small but significant indirect effects on changes in HLA via parental stress. The indirect effects were as follows: β_ind_ = –0.00 (*SE* = 0.00, *p* = 0.269) for financial problems, β_ind_ = –0.02 (*SE* = 0.00, *p* = 0.000) for problematic housing, β_ind_ = –0.04 (*SE* = 0.00, *p* = 0.001) for work-related problems, β_ind_ = –0.01 (*SE* = 0.00, *p* = 0.002) for health worries, β_ind_ = –0.03 (*SE* = 0.00, *p* = 0.000) for conflict with partner and β_ind_ = –0.03 (*SE* = 0.00, *p* = 0.000) for conflict with family.

**TABLE 3:** Regression results for stressors and perceived stress predicting changes in HLA.

	Model 1			Model 2			Model 3		
			
	β	*SE*	*p*	β	*SE*	*p*	β	*SE*	*p*
***Covariates***									
Gender (0 = female)	0.02	0.01	0.091	0.02	0.01	0.135	0.02	0.01	0.177
Age in years	–0.06	0.01	0.000	–0.05	0.01	0.000	–0.05	0.01	0.000
Education level	0.04	0.01	0.001	0.04	0.01	0.002	0.04	0.01	0.003
Age of child in years	–0.07	0.02	0.000	–0.08	0.02	0.000	–0.08	0.02	0.000
No. of children ages 1–6	–0.05	0.02	0.001	–0.04	0.01	0.016	–0.03	0.01	0.025
Single parent (0 = no)	0.00	0.01	0.867	0.01	0.01	0.622	0.01	0.01	0.472
Private childcare (0 = no)	–0.05	0.02	0.005	–0.05	0.02	0.002	–0.05	0.02	0.002
Working from home (0 = no)	–0.00	0.02	0.955	0.02	0.02	0.212	0.02	0.02	0.200
Both partners working (0 = no)	–0.03	0.01	0.024	–0.02	0.01	0.116	–0.02	0.01	0.205
***Stressors***									
Financial problems	–0.05	0.02	0.001	–0.05	0.02	0.001	–0.05	0.02	0.001
Problematic housing	–0.01	0.01	0.565	0.02	0.01	0.267	0.02	0.01	0.231
Work-related problems	–0.03	0.01	0.022	0.00	0.01	0.768	0.00	0.01	0.773
COVID-related health worries	0.08	0.01	0.000	0.08	0.01	0.000	0.08	0.01	0.000
Conflict with partner	–0.01	0.01	0.459	0.02	0.01	0.176	0.02	0.01	0.178
Conflict with family	–0.04	0.01	0.003	–0.01	0.02	0.480	–0.01	0.02	0.665
***Stress***									
Parental stress				–0.16	0.01	0.000	0.05	0.04	0.128
*Exp*(parental stress)							–0.23	0.04	0.000
*R* ^2^	0.03	0.01	0.000	0.05	0.01	0.000	0.06	0.01	0.000

**N* = 7,837. *exp*(parental stress), exponentiated parental stress term.*

**FIGURE 1:**
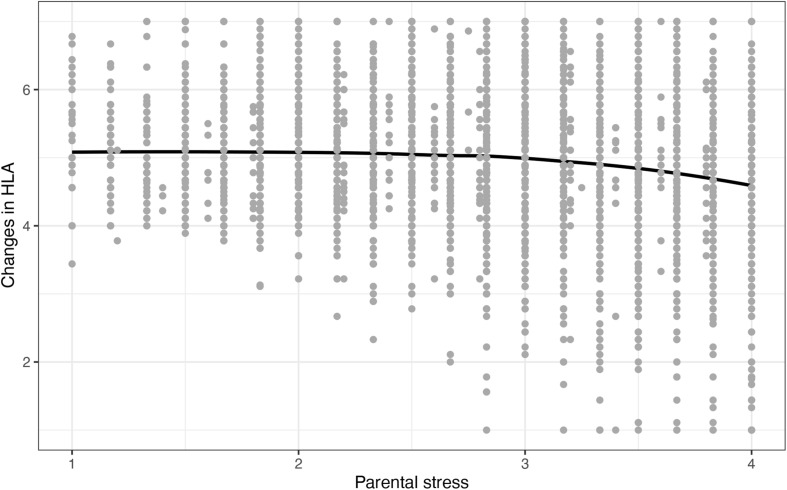
LOESS curve showing the relation between perceived parental stress and changes in HLA.

In the third step, we tested the threshold hypothesis stating that stress is non-linearly related to changes in HLA (H1c). We started by visually examining the relation between perceived parental stress and HLA using locally estimated scatterplot smoothing (LOESS), see Figure 1. The LOESS curve revealed that HLA remained relatively unaffected by parental stress until a tipping point is reached around a stress score of about three on the original scale, ranging from 1 (*totally disagree*) to 4 (*totally agree*). Above this tipping point, changes in HLA decreased with increasing stress. The shape of the LOESS curve suggested an exponential negative relation between stress and changes in HLA. Based on this finding, we included an exponentiated stress score in the hierarchical regression model (see Table 3, Model 3). Results showed a significant negative effect of the exponentiated stress term and a small increase in the amount of explained variance compared to Model 2 (Δ*R*^2^ = 0.01)^5^. Taken together, the LOESS plot and the results of the hierarchical regression analyses showed that HLA exponentially decreased with increasing stress. Although the average HLA score remained above four (=same HLA as before the lockdown), there was particularly high variance in HLA among the very stressed parents (see distribution of cases, indicated by dots in Figure 1). Thus, some of the very stressed parents reported to offer less HLA than before the lockdown – which was not the case among parents who were not stressed.

### RQ2: Role of Protective Factors Parental Self-Efficacy and Perceived Social Support

Our second research question tackled the role of parental self-efficacy and perceived social support in the relation between parental stress and changes in HLA. Our hypotheses stated that parental self-efficacy and perceived social support are negatively related to parental stress (H2a) and positively related to changes in HLA (H2b). We tested these assumptions using path analyses. We controlled for all covariates in the model. The overall model fit was excellent (χ^2^ = 56.05, *df* = 6, RMSEA = 0.03, CFI = 0.99, TLI = 0.98, SRMR = 0.01). Results are displayed in Table 4. In line with our hypothesis 2a, parental self-efficacy and perceived social support were negatively related to perceived stress, i.e., the higher parents’ self-efficacy beliefs and the more they felt supported, the lower their stress level. The model explained more variance in parental stress than the regression model without parental self-efficacy and perceived social support (Δ*R*^2^ = 0.08, see Table 2). Our hypothesis 2b could partly be supported: Perceived social support, but not parental self-efficacy beliefs, was positively related to changes in HLA: The higher parents’ perceived social support, the more HLA they offered compared to before the lockdown.

**TABLE 4:** Relation between parental self-efficacy, perceived support, perceived stress, and changes in HLA.

	Parental stress			Changes in HLA		
		
	β	*SE*	*p*	β	*SE*	*p*
***Covariates***						
Gender (0 = female)	–0.04	0.01	0.000	0.02	0.01	0.048
Age in years	0.00	0.01	0.928	–0.05	0.01	0.001
Education level	–0.01	0.01	0.621	0.04	0.01	0.001
Age of child in years	–0.05	0.01	0.000	–0.08	0.02	0.000
No. of children ages 1–6	0.07	0.01	0.000	–0.04	0.01	0.010
Single parent (0 = no)	0.01	0.01	0.322	0.01	0.01	0.576
Private childcare (0 = no)	–0.01	0.02	0.559	–0.06	0.02	0.000
Working from home (0 = no)	0.12	0.01	0.000	0.02	0.01	0.099
Both partners working (0 = no)	0.06	0.01	0.000	–0.02	0.01	0.097
***Stressors***						
Financial problems	0.00	0.01	0.702			
Problematic housing	0.11	0.01	0.000			
Work-related problems	0.19	0.01	0.000			
COVID-related health worries	0.03	0.01	0.002			
Conflict with partner	0.13	0.01	0.000			
Conflict with family	0.16	0.01	0.000			
***Predictors***						
Parental stress				0.10	0.03	0.004
*Exp*(parental stress)				–0.23	0.04	0.000
Parental self-efficacy	–0.20	0.01	0.000	0.02	0.01	0.094
Perceived support	–0.19	0.01	0.000	0.07	0.01	0.000
*R* ^2^	0.39	0.01	0.000	0.05	0.01	0.000

**N* = 7,837.*

**FIGURE 2:**
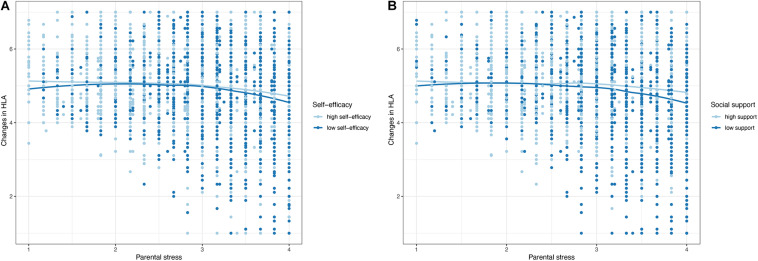
LOESS plots for the relation between perceived parental stress and changes in HLA moderated by **(A)** parental self-efficacy and **(B)** perceived support.

Although parental self-efficacy and perceived social support were not strong predictors of changes in HLA, they may nevertheless buffer the negative influence of stress on changes in HLA (hypothesis 2c). We first visually examined whether the relation between stress and changes in HLA varied for parents with low versus high self-efficacy (Figure 2A) or low versus high perceived social support (Figure 2B). Median-split was used to compare low (0) and high (1) parental self-efficacy/perceived social support groups in their LOESS-curves in R (Wickham, 2016; RStudio Team, 2020). The LOESS curves for parents with low versus high parental self-efficacy were very similar, indicating only a small interaction (see Figure 2A). The LOESS curves for low versus high perceived social support differed more strongly: parents with low perceived social support showed a steeper decrease in HLA with increased stress than parents with high perceived social support.

Based on this finding, we tested for statistical interaction in a multivariate regression model. The model included (a) main effects of parental stress, parental self-efficacy, and perceived social support predicting changes in HLA as well as (b) the interaction terms of parental self-efficacy × parental stress, and perceived social support × parental stress. Because the relation between stress and changes in HLA was exponential, we report average marginal effects (AME) (Mize, 2019), which refer to the average change in HLA for one unit (*SD*) change in stress, at different values of self-efficacy/perceived social support. We chose to examine the AME of stress on changes in HLA at the 0–100% quartiles of self-efficacy/perceived social support, in order to determine whether the AME of stress on HLA varies depending on parents’ self-efficacy and/or social support score. The results are displayed in Table 5. The negative AME of stress on changes in HLA continuously decreased with increasing self-efficacy, indicating that parental self-efficacy partly buffered the negative effect of stress on HLA. However, the negative AME for the 100% self-efficacy percentile indicated that even high parental self-efficacy beliefs could not completely buffer the negative effect of stress on HLA (indicated by the significant negative relation between stress and changes in HLA at the 100% self-efficacy percentile). For perceived social support, results showed a similar but stronger trend: The negative effect of stress on changes in HLA became weaker with increasing perceived social support. For parents with very high perceived social support values (100% percentile), the negative effect of stress even became non-significant, indicating that perceived social support could completely buffer the negative effect of stress on changes in HLA. Thus, parental self-efficacy and – to a larger extend – perceived social support interacted with parental stress in relation to changes in HLA.

**TABLE 5:** Average marginal effects of stress on changes in HLA at different percentiles of parental self-efficacy and perceived social support.

	AME	*SE*	*p*	95% CI
***AME of stress on changes in HLA at self-efficacy percentiles (associated self-efficacy raw scores):***				
0% percentile (1.00)	–0.29	0.07	0.000	[–0.43; –0.15]
25% percentile (2.25)	–0.17	0.02	0.000	[–0.20; –0.14]
50% percentile (2.75)	–0.16	0.01	0.001	[–0.19; –0.13]
75% percentile (3.25)	–0.13	0.02	0.000	[–0.17; –0.10]
100% percentile (4.00)	–0.11	0.03	0.000	[–0.16; –0.06]
***AME of stress on changes in HLA at support percentiles (associated social support raw scores):***				
0% percentile (1.00)	–0.30	0.04	0.000	[–0.37; –0.23]
25% percentile (2.50)	–0.20	0.01	0.000	[–0.24; –0.16]
50% percentile (3.25)	–0.15	0.01	0.000	[–0.18; –0.12]
75% percentile (4.00)	–0.10	0.02	0.000	[–0.13; –0.06]
100% percentile (5.00)	–0.03	0.03	0.266	[–0.08; 0.02]

**N* = 7,837. AME, average marginal effect; *SE*, standard error; 95% CI, 95% Confidence intervals.*

## Discussion

The present study was one of the first in Germany to examine parental stress and changes in parents’ HLA during the COVID-19 lockdown in Spring 2020. In addition, we considered the role of potential protective factors in the relation between parental stress and changes in HLA. Our key findings can be summarized as follows: Parents engaged, on average, in more HLA with their children compared to before the lockdown. Parental stress predicted self-reported changes in HLA: The lower parents’ stress, the higher the increase in HLA. This relation, however, was non-linear and showed an exponential decline in HLA starting at an above-average stress score. Thus, whereas most parents offered more HLA, some of the very stressed parents offered less HLA than before the lockdown. Parental self-efficacy and perceived social support were protective of parental stress, i.e., parents with higher parental self-efficacy and an intact social support system experienced less stress during the lockdown. In addition, we found significant interaction effects of self-efficacy and perceived social support with stress in relation to changes in HLA. In the following, we discuss these findings as well as the implications for research and practice.

### Changes in Home Learning Activities During the COVID-19 Lockdown

The cognitively stimulating HLA that parents provide are crucial for child development (Tietze et al., 1998; NICHD ECCRN, 2003; Anders et al., 2012; Kluczniok et al., 2013). The temporary closure of daycare centers during the COVID-19 lockdown amplified the importance of HLA: Children were deprived of all other forms of early childhood education and other play opportunities, including institutional education in preschools, educational programs or extracurricular activities. Thus, more than ever, children relied on parents’ ability to provide a stimulating learning environment at home. The results of our study showed that parents, on average, reported to do *slightly more* HLA compared to before the lockdown on a 7-point scale ranging *much less* to *much more*. However, there was a lot a variance in HLA, indicating that not all parents offered more HLA. These changes of HLA during the COVID-19 lockdown in comparison to before the lockdown could be explained by a number of COVID-19-related influences, one of which may be increased perceived stress. Specifically, the COVID-19 lockdown likely created a particularly challenging situation for parents, which increased their stress and may have undermined some parents’ ability to offer HLA.

### Relation Between Stressors, Parental Stress, and Changes in HLA

The family stress model proposes that stressors increase parental stress, which undermines supportive parenting, including parents’ ability to offer HLA (Gershoff et al., 2007; Raver et al., 2007). In line with these assumption, results of the present study revealed that stressors, including work-related problems, conflict with family, and conflict with partner, were positively related to perceived parental stress during the COVID-19 lockdown: The more the parents rated these stressors as burdensome, the more stress they reported. Parental stress was – in turn – negatively related to changes in parents’ provision of HLA. This finding is in line with previous research documenting a negative relation between parental stress and parenting practices, including HLA (Gershoff et al., 2007; Wolf et al., 2019; Bendickson, 2020). Importantly, these relations were found after accounting for a number of relevant family characteristics, including parents’ gender, age and education, the age and number of children, single parents and parents’ occupational status. Moreover, the results were controlled for the influence of parents’ financial status, which may have been associated with perceived stress and/or parents’ financial resources for home learning materials. Similarly, we controlled for private childcare and whether parents were working from home because these covariates may have been associated with perceived stress and/or time available for HLA. The associations found between stress and changes in HLA were thus independent of child and family background characteristics (including financial problems) as well as distal indicators of the time available to spend with their children (e.g., private childcare and working from home).

Analyses further showed that the relation between parental stress and changes in HLA was non-linear: there was no relation between perceived parental stress and changes in HLA among parents with a stress score below three on the original scale, ranging from 1 (*totally disagree*) to 4 (*totally agree*). Past this tipping point, HLA exponentially decreased with increasing stress. To our knowledge, this was the first study to test for and reveal a non-linear relation between parental stress and one aspect of parenting behavior, namely changes in parents’ provision of HLA. This finding has important theoretical implications for the family stress model (Conger et al., 1992; Masarik and Conger, 2017), which assumes a linear relation between parental stress and parenting practices. Based on our results we argue that it is necessary to question this assumption and test for a potential non-linear association between parental stress and parenting practices in future research.

Another interesting result of our study was the high variance in changes of HLA among parents with higher stress scores. In fact, although most parents – on average – reported to offer slightly more HLA than before the lockdown, there were a number of parents who reported to offer (much) less HLA than before the lockdown, which did not occur among parents who were less stressed. Based on these findings, we considered additional protective factors that may help explain why some parents were able to offer more HLA than before the lockdown despite their high stress scores whereas others were not.

### Role of Protective Factors: Parental Self-Efficacy and Perceived Social Support

It has been proposed that the negative associations between stress and parenting behavior can be disrupted by protective factors (Masarik and Conger, 2017). Previous studies have shown that high parental self-efficacy beliefs and a supportive social network can reduce parental stress and improve parenting behavior (Green et al., 2007; McConnell et al., 2011; Bojczyk et al., 2018). In line with previous findings (Östberg and Hagekull, 2000; McConnell et al., 2011), results of the present study showed that self-efficacy beliefs and perceived social support were both protective of parental stress. In addition, results showed significant interaction effects of parental self-efficacy and perceived social support with stress in the relation to changes in HLA. Our finding of main and interaction effects of the two protective factors is in accordance with the assumptions of the adapted family stress model by Masarik and Conger (2017). Specifically, high parental self-efficacy and an intact social support system seemed to buffer the negative influence of existing stress on changes in HLA. This buffering effect was stronger for perceived social support: Whereas high parental self-efficacy beliefs were only able to *reduce* the negative influence of stress on changes in HLA, high perceived social support *eliminated* the negative relation between stress and changes in HLA. Thus, parental self-efficacy and – to a larger degree – perceived social support seemed to help parents to provide more HLA during the COVID-19 lockdown despite their stress. The mechanisms behind this finding may be that parents who are more confident in their ability to support their children’s development even in challenging times and parents who can rely upon emotional and informational support from their social network, are better at coping with challenging situations, such as the COVID-19 lockdown, and consequently offer more HLA despite their stress.

### Limitations

There are several limitations to the present study that should be noted. First, recruitment of study participants was based on convenience sampling and participation in the study was optional. Thus, although the sample was very large and drawn from all states of Germany, it was not a random selection, resulting in selection bias. For instance, parents with low educational levels and single parents were underrepresented in our sample compared to the German average (Statistisches Bundesamt, 2020). In order to minimize the influence of these potential biases, we controlled for a number of family’s background characteristics in our analyses (e.g., parents’ age, gender and education, single parent, children’s age, number of children ages 1–6, single or dual earner households).

Second, we were unable to infer causal relations from the cross-sectionally examined variables. As previous research has suggested bi-directional relations between some of the study variables, e.g., parental stress and self-efficacy (Crnic and Ross, 2017), future longitudinal studies should test the directionality of the effects. Moreover, due to the cross-sectional nature of the study, we could only test for statistical interaction effects as testing for unidirectional moderation requires longitudinal data (Hall et al., 2020).

Third, due to the cross-sectional data, changes in HLA could not be directly tested. As we anticipated this limitation, we directly asked parents to indicate the extent to which HLA has changed compared to before the lockdown. This allowed us to investigate how parents’ provision of HLA at home changed as a result of the COVID-19 lockdown – and to examine the role of parental stress, self-efficacy, and perceived social support for these reported changes in HLA. However, this approach also meant that we do not have any information about parents’ baseline frequency of HLA. Thus, our study results can be interpreted in terms of how parental stress, self-efficacy, and perceived social support affected changes in HLA, but we cannot generalize these findings to absolute HLA frequencies. Related to this limitation, it is also important to note that our HLA measure captures changes in HLA that parents engaged in with their children, compared to before the lockdown. It does not, however, represent changes in children’s overall learning opportunities at home and at preschool, which were likely much lower due to the closure of institutional daycare.

Lastly, the HLA measure relied on parental self-report which may be biased. Although we cannot exclude that social desirability influenced parents’ self-reported changes in HLA, it seems unlikely given the high variance of self-reported changes in HLA. Moreover, social desirability bias would only affect the generalizability of the central tendencies of our HLA measure but not the generalizability of the found associations.

## Conclusion and Implications

The results of the present study showed that parents, on average, provided more HLA than before the lockdown. Thus, stressful situations, such as the COVID-19 lockdown, do not seem to automatically translate into a decrease of HLA for young children, which is good news for practice and policy. Instead, the amount of parental stress seemed to matter for parents’ provision of HLA: while little stress had no effect on changes in HLA, once parents reached a certain stress level, changes in HLA exponentially decreased with increasing stress. The implications for research and practice are that more attention should be paid to these parents who were very stressed. In this regard, the present study provides the important insights that the harmful effects of parental stress on changes in HLA could be buffered by high parental self-efficacy and perceived social support. Thus, measures should be undertaken to promote parental self-efficacy and provide social support particularly during stressful times, such as the COVID-19 lockdown. This could be achieved through (digital) family support initiatives, e.g., digital play groups to foster exchange among parents, platforms that enable exchange among parents and preschool staff as well as the provision of ideas and materials for HLA. In addition, existing family support programs could be extended to be accessed digitally. These family support initiatives could reduce parental stress and help parents provide HLA for their children despite the challenging circumstances. However, it is important to keep in mind that this cannot compensate for the lack of institutional education and care. Closing daycare centers are extreme measures that deprive children of the education and the social contact that they need while putting parents under immense stress. This can be particularly harmful for families living in disadvantageous circumstances. Thus, the closure of institutional education and care should be a last resort and only be implemented for short times, since these measures take the largest toll on families with young children.

## Data Availability Statement

The datasets presented in this article are not readily available because the data are currently reserved for scientific qualifications (Ph.D. and masters’ theses). Requests to access the datasets should be directed to EO, elisa.oppermann@uni-bamberg.

## Ethics Statement

Ethical review and approval was not required for the study on human participants in accordance with the local legislation and institutional requirements. The patients/participants provided their written informed consent to participate in this study.

## Author Contributions

EO: conceptualization, data collection, methodology, formal analysis, writing – original draft, and visualization. FC: conceptualization, data collection, and writing – review and editing. KW and LB: conceptualization, methodology, and writing – review and editing. YA: conceptualization, writing – review and editing, supervision, and resources. All authors contributed to the article and approved the submitted version.

## Conflict of Interest

The authors declare that the research was conducted in the absence of any commercial or financial relationships that could be construed as a potential conflict of interest.

## Publisher’s Note

All claims expressed in this article are solely those of the authors and do not necessarily represent those of their affiliated organizations, or those of the publisher, the editors and the reviewers. Any product that may be evaluated in this article, or claim that may be made by its manufacturer, is not guaranteed or endorsed by the publisher.

## Footnotes

1 Emergency childcare was only available to a small number of families in systemically relevant occupations.

2 In this article, we refer to this time of strict restrictions in Germany between March and May as “lockdown.” Although the restrictions in Germany were – strictly speaking – not a lockdown in the literal sense because curfews were only implemented in some states, the restrictions limited individuals’ lives severely and are typically referred to as “lockdown” in the media as well as in previous publications (e.g., Huebener et al., 2021).

3 In Germany, parents with system relevant occupations were eligible to emergency childcare, often for a reduced number of hours.

4
www.dfg.de/en/research_funding/faq/faq_humanities_social_science/index.html

5 Additional multigroup analyses in Mplus were conducted to examine whether the strength of the exponential relation between parental stress and changes in HLA differed between children’s age groups (1–2-year-olds, 3–4-year-olds, and 5–6-year-olds). Although the relation between parental stress and changes in HLA was slightly lower among parents of 1–2-year-olds than among parents of 3–4-year-olds and 5–6-year-olds (see Supplementary Appendix Table A5), these small differences were not significant (*p* > 0.05).
