# Supplementary material for: Changes in Parents’ Home Learning Activities With Their Children During the COVID-19 Lockdown – The Role of Parental Stress, Parents’ Self-Efficacy and Social Support
Source: Front Psychol. 2021 Jul 29;12:682540. doi: 10.3389/fpsyg.2021.682540 (PMC8359822; doi:10.3389/fpsyg.2021.682540)
Supplement: Supplementary file 1 [file Table_1.docx]

# Appendix

**Table A1**

Item Wordings and Descriptive Statistics for HLA Items

|  | M | SD |
| --- | --- | --- |
| Reading a (picture) book together with the child | 5.00 | 1.27 |
| Collecting, sorting and comparing objects | 4.82 | 1.23 |
| Playing board games or puzzles | 5.02 | 1.30 |
| Building- and construction games | 4.94 | 1.24 |
| Artistic creative activity, e.g., painting or handicrafts | 5.61 | 1.27 |
| Singing, making music, dancing | 5.11 | 1.25 |
| Teaching the child rhymes, songs or poems | 4.23 | 1.14 |
| Role playing games | 4.56 | 1.28 |
| Motion play (e.g., running, playing tag, hide, and seek) | 5.34 | 1.35 |

*Note.* *N* = 7,837. Response formats ranged from *much less* (1), *less* (2), *slightly less* (3), *same* (4), *slightly more* (5), *more* (6) to *much more* (7) than before the lockdown.
