# Supplementary material for: Changes in Parents’ Home Learning Activities With Their Children During the COVID-19 Lockdown – The Role of Parental Stress, Parents’ Self-Efficacy and Social Support
Source: Front Psychol. 2021 Jul 29;12:682540. doi: 10.3389/fpsyg.2021.682540 (PMC8359822; doi:10.3389/fpsyg.2021.682540)
Supplement: Supplementary file 2 [file Table_2.docx]

**Table A2**

Item Wordings for Parents’ Perceived Support

|  |
| --- |
| Can you rely upon someone to give you advice with problems? |
| Can you count on someone to provide you with emotional support? |
| Do you have someone with whom you can discuss parenting issues? |
| Do you generally feel supported by your social network? |
