# Supplementary material for: Changes in Parents’ Home Learning Activities With Their Children During the COVID-19 Lockdown – The Role of Parental Stress, Parents’ Self-Efficacy and Social Support
Source: Front Psychol. 2021 Jul 29;12:682540. doi: 10.3389/fpsyg.2021.682540 (PMC8359822; doi:10.3389/fpsyg.2021.682540)
Supplement: Supplementary file 3 [file Table_3.docx]

**Table A3**

Descriptive Statistics by Age Group

|  | 1-2-year-olds | | 3-4-year-olds | | | 5-6-year-olds | | |  |
| --- | --- | --- | --- | --- | --- | --- | --- | --- | --- |
|  | M / % | SD | | M / % | SD | | M / % | SD | |
| No of children ages 1-6 | 1.41_a_ | 0.61 | | 1.48_a_ | 0.54 | | 1.65_a_ | 0.62 | |
| Single parent in % | 3.1% _ab_ |  | | 4.0% _a_ |  | | 5.0% _b_ |  | |
| Private childcare in % | 27.0% _ab_ |  | | 22.8% _a_ |  | | 21.9% _b_ |  | |
| Working from home in % | 73.8% _ab_ |  | | 72.8% _a_ |  | | 72.7% _b_ |  | |
| Both partners working in % | 72.1% _a_ |  | | 58.6% _a_ |  | | 68.6% _a_ |  | |
| Financial problems | 2.23 | 1.21 | | 2.15 | 1.17 | | 2.15 | 1.19 | |
| Problematic housing | 2.23_a_ | 1.15 | | 2.20_b_ | 1.16 | | 2.07_ab_ | 1.10 | |
| Work-related problems | 3.17_ab_ | 1.23 | | 2.99_a_ | 1.27 | | 3.00_b_ | 1.28 | |
| COVID-related health worries | 2.64 | 1.23 | | 2.65 | 1.23 | | 2.59 | 1.23 | |
| Conflict with partner | 2.63_a_ | 1.15 | | 2.63_b_ | 1.13 | | 2.55_ab_ | 1.27 | |
| Conflict with family | 2.24_ab_ | 1.14 | | 2.27_a_ | 1.10 | | 2.32_b_ | 1.10 | |
| Parental stress | 2.71 | 0.74 | | 2.72 | 0.71 | | 2.69 | 0.73 | |
| Changes in HLA | 4.98_a_ | 0.89 | | 5.03_b_ | 0.82 | | 4.89_ab_ | 0.82 | |
| Parental self-efficacy | 3.27_ab_ | 0.43 | | 3.24_a_ | 0.43 | | 3.21_b_ | 0.44 | |
| Perceived social support | 3.33 | 1.03 | | 3.34 | 1.01 | | 3.31 | 1.02 | |
| N | 1,787 |  | | 2,812 |  | | 3,238 |  | |

*Note*. We used the Tamhane T2 posthoc test which is robust to unequal samples sized and variances across groups to test for mean differences. Means within a row sharing the same subscripts are significantly different at the *p* < .05 level.
