# Supplementary material for: Changes in Parents’ Home Learning Activities With Their Children During the COVID-19 Lockdown – The Role of Parental Stress, Parents’ Self-Efficacy and Social Support
Source: Front Psychol. 2021 Jul 29;12:682540. doi: 10.3389/fpsyg.2021.682540 (PMC8359822; doi:10.3389/fpsyg.2021.682540)
Supplement: Supplementary file 4 [file Table_4.docx]

**Table A4**

Bivariate Correlations

|  |  | 1 | 2 | 3 | 4 | 5 | 6 | 7 | 8 | 9 | 10 | 11 | 12 | 13 | 14 | 15 | 16 |
| --- | --- | --- | --- | --- | --- | --- | --- | --- | --- | --- | --- | --- | --- | --- | --- | --- | --- |
| 1 | Age of child |  |  |  |  |  |  |  |  |  |  |  |  |  |  |  |  |
| 2 | No of children | .27^***^ |  |  |  |  |  |  |  |  |  |  |  |  |  |  |  |
| 3 | Single parent | .05^***^ | -.10^***^ |  |  |  |  |  |  |  |  |  |  |  |  |  |  |
| 4 | Private childcare | -.05^**^ | -.06^***^ | .08^***^ |  |  |  |  |  |  |  |  |  |  |  |  |  |
| 5 | Working from home | -.04^**^ | -.03 | .01 | -.16^***^ |  |  |  |  |  |  |  |  |  |  |  |  |
| 6 | Both partners working | .00 | -.30^***^ | -.23^***^ | .11^***^ | .05^*^ |  |  |  |  |  |  |  |  |  |  |  |
| 7 | Financial problems | -.01 | .00 | .09^***^ | .02 | -.16^***^ | -.10^***^ |  |  |  |  |  |  |  |  |  |  |
| 8 | Problematic housing | -.01^***^ | .00 | .04^**^ | -.02 | .03^*^ | -.06^***^ | .31^***^ |  |  |  |  |  |  |  |  |  |
| 9 | Work-related problems | -.05^***^ | -.10^***^ | .02 | -.01 | .04^**^ | .14^***^ | .39^***^ | .32^***^ |  |  |  |  |  |  |  |  |
| 10 | COVID-related health worries | -.03^**^ | -.04^**^ | .03 | -.04^**^ | .03^*^ | -.03^*^ | .21^***^ | .17^***^ | .17^***^ |  |  |  |  |  |  |  |
| 11 | Conflict with partner | -.04^***^ | .02 | -.13^***^ | -.02 | .05^***^ | .01 | .20^***^ | .27^***^ | .25^***^ | .15^***^ |  |  |  |  |  |  |
| 12 | Conflict with family | .04^**^ | .07^***^ | -.03^*^ | .00 | .03 | -.03^*^ | .18^***^ | .25^***^ | .23^***^ | .15^***^ | .49^***^ |  |  |  |  |  |
| 13 | Cumulated stressor index | -.05^***^ | -.00 | -.01 | -.02 | .00 | -.02 | .54^***^ | .57^***^ | .58^***^ | .45^***^ | .59^***^ | .58^***^ |  |  |  |  |
| 14 | Parental stress | -.04^**^ | .03^**^ | -.01 | -.05^**^ | .15^***^ | .06^***^ | .20^***^ | .32^***^ | .38^***^ | .16^***^ | .39^***^ | .39^***^ | .46^***^ |  |  |  |
| 15 | Changes in HLA | -.10^***^ | -.05^***^ | .00 | -.05^**^ | .02 | -.03^*^ | -.06^***^ | -.02 | -.05^***^ | .06^***^ | -.04^**^ | -.06^**^ | -.03^*^ | -.14^***^ |  |  |
| 16 | Parental self-efficacy | -.05^***^ | -.04^***^ | -.03^*^ | .00 | -.02 | .02 | -.09^***^ | -.12^***^ | -.11^***^ | -.07^***^ | -.20^***^ | -.20^***^ | -.22^***^ | -.34^***^ | .08^***^ |  |
| 17 | Perceived social support | -.01 | -.01 | -.01 | .12^***^ | -.04^**^ | .02 | -.16^***^ | -.23^***^ | -.22^***^ | -.08^***^ | -.26^***^ | -.24^***^ | -.30^***^ | -.38^***^ | .11^***^ | .23^***^ |

*Note.* *N* = 7,837. * *p* < .05, ** *p* < .01; *** *p* < .001.
