# Supplementary material for: Changes in Parents’ Home Learning Activities With Their Children During the COVID-19 Lockdown – The Role of Parental Stress, Parents’ Self-Efficacy and Social Support
Source: Front Psychol. 2021 Jul 29;12:682540. doi: 10.3389/fpsyg.2021.682540 (PMC8359822; doi:10.3389/fpsyg.2021.682540)
Supplement: Supplementary file 5 [file Table_5.docx]

**Table A5**

Regression Results for Stressors and Perceived Stress Predicting Changes in HLA for Different Age Groups

| *Child ages* | *1-2-year-olds* | | | | | | *3-4-year-olds* | | | | | | | *5-6-year-olds* | | | | | | |  |  |
| --- | --- | --- | --- | --- | --- | --- | --- | --- | --- | --- | --- | --- | --- | --- | --- | --- | --- | --- | --- | --- | --- | --- |
|  | β | | *SE* | | *p* | | | β | | *SE* | | *p* | | | β | | *SE* | | *p* | | |  |
| *Covariates* | |  | |  | |  | | |  | |  | |  | | |  | |  | |  | | |
| Gender (0 = female) | | .06 | | .02 | | .012 | | | -.02 | | .02 | | .389 | | | .02 | | .02 | | .192 | | |
| Age in years | | -.08 | | .03 | | .003 | | | -.06 | | .02 | | .005 | | | -.04 | | .02 | | .065 | | |
| Education level | | .03 | | .03 | | .290 | | | -.01 | | .02 | | .006 | | | .03 | | .02 | | .156 | | |
| Age of child in years | | .07 | | .04 | | .097 | | | -.01 | | .02 | | .714 | | | -.04 | | .02 | | .030 | | |
| No of children ages 1-6 | | -.09 | | .03 | | .003 | | | -.09 | | .02 | | .000 | | | .01 | | .02 | | .544 | | |
| Single parent (0 = no) | | .00 | | .03 | | .956 | | | .00 | | .02 | | .891 | | | .01 | | .02 | | .466 | | |
| Private childcare (0 = no) | | -.04 | | .04 | | .295 | | | -.08 | | .03 | | .004 | | | -.03 | | .03 | | .245 | | |
| Working from home (0 = no) | | .02 | | .03 | | .476 | | | .05 | | .02 | | .048 | | | -.00 | | .02 | | .913 | | |
| Both partners working (0 = no) | | -.03 | | .03 | | .263 | | | -.03 | | .02 | | .280 | | | -.01 | | .02 | | .598 | | |
| *Stressors* | |  | |  | |  | | |  | |  | |  | | |  | |  | |  | | |
| Financial problems | | -.05 | | .03 | | .113 | | | -.05 | | .02 | | .047 | | | -.05 | | .02 | | .035 | | |
| Problematic housing | | .03 | | .03 | | .348 | | | .02 | | .02 | | .302 | | | .01 | | .02 | | .748 | | |
| Work-related problems | | .01 | | .03 | | .670 | | | -.00 | | .02 | | .912 | | | .00 | | .02 | | .863 | | |
| COVID-related health worries | | .05 | | .03 | | .062 | | | .09 | | .02 | | .000 | | | .09 | | .02 | | .000 | | |
| Conflict with partner | | .01 | | .03 | | .649 | | | .04 | | .02 | | .088 | | | .00 | | .02 | | .873 | | |
| Conflict with family | | -.01 | | .03 | | .881 | | | .00 | | .02 | | .879 | | | -.02 | | .02 | | .494 | | |
| *Stress* | |  | |  | |  | | |  | |  | |  | | |  | |  | |  | | |
| Parental stress | | .00 | | .08 | | .988 | | | .09 | | .06 | | .144 | | | .04 | | .05 | | .395 | | |
| *exp*(parental stress) | | -.18 | | .09 | | .044 | | | -.26 | | .06 | | .000 | | | -.23 | | .06 | | .000 | | |
| *R*^2^ | | .06 | | .01 | | .000 | | | .06 | | .01 | | .000 | | | .05 | | .01 | | .000 | | |
| *N* | | 1,787 | | | | | | | 2,812 | | | |  | | | 3,238 | | | | | | |

*Note*. Total *N* = 7,837. *exp*(parental stress) = exponentiated parental stress term.
